# Andersen-Tawil syndrome: visual clues to the diagnosis

**DOI:** 10.1055/s-0046-1820534

**Published:** 2026-05-12

**Authors:** João Victor Cabral Correia Férrer, Gabriel Keller Guimarães, Stefano Machado, Carlos Otto Heise, Andre Macedo Serafim da Silva, Edmar Zanoteli

**Affiliations:** 1 Universidade de São Paulo, Faculdade de Medicina, Departamento de Neurologia, São Paulo SP, Brazil.

**Figure 1:**
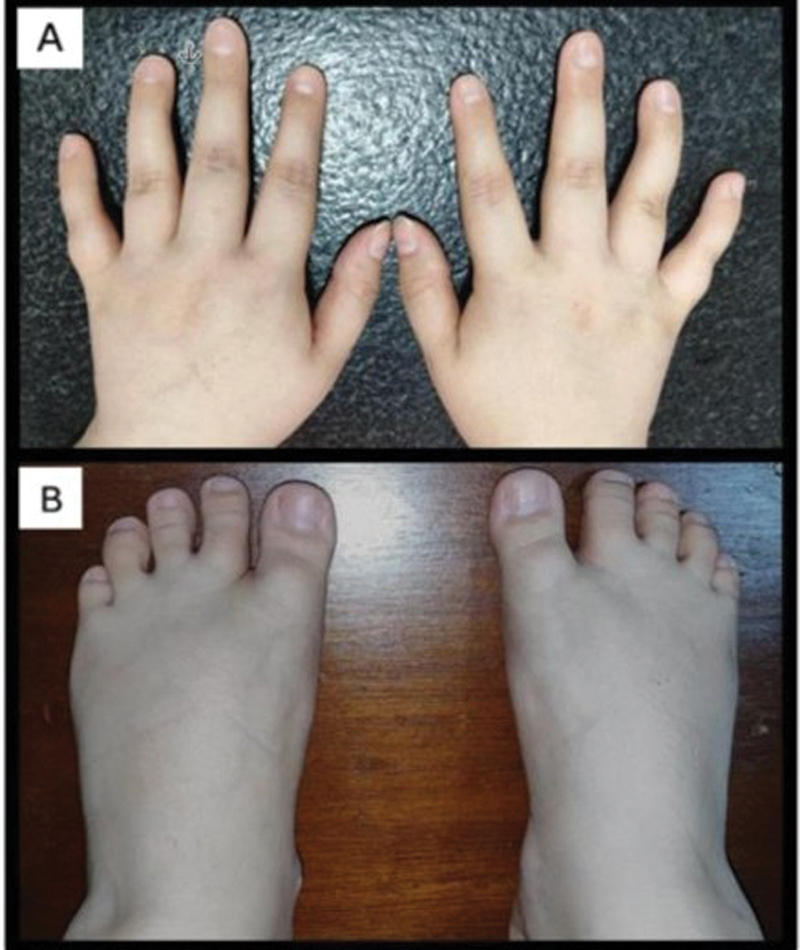
( **A** ) Bilateral fourth-finger clinodactyly. ( **B** ) Bilateral syndactyly between the second and third toes.

**Figure 2:**
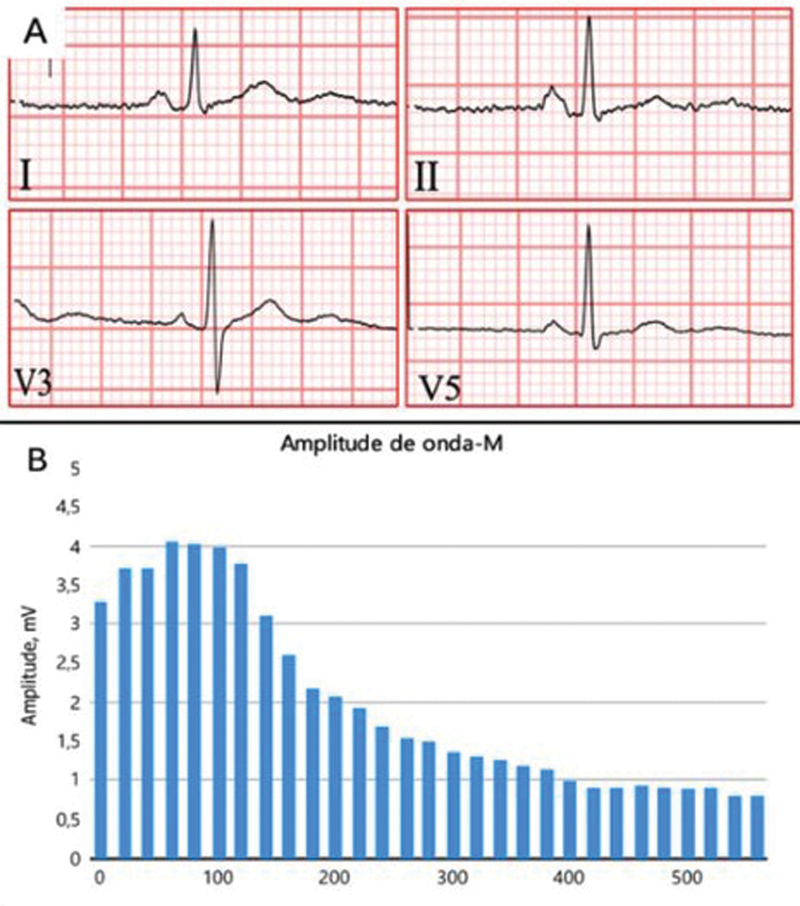
( **A** ) Electrocardiogram: Prolonged QT interval and U waves. ( **B** ) Abnormal prolonged exercise test: Initial compound muscle action potential (CMAP) amplitude increment of 23%, followed by 78% CMAP amplitude decrement in 50 minutes.

We report the case of a 26-year-old woman with long QT syndrome and recurrent episodes of muscle weakness with onset in childhood, lasting from hours to days, and a similar paternal family history. An examination revealed micrognathia and clinodactyly ( Figure 1 ). The prolonged exercise test showed an initial increase followed by a 78% reduction in the amplitude of the compound muscle action potential (CMAP) ( Figure 2 ). Exome sequencing identified a likely pathogenic heterozygous variant in the *potassium inwardly rectifying channel subfamily J member 2* ( *KCNJ2* ) gene (p.Ile176_Gly177insGlyIle), confirming Andersen-Tawil syndrome (ATS).

The syndrome is an autosomal dominant disorder characterized by a clinical triad of craniofacial and skeletal dysmorphism, periodic paralysis, and ventricular arrhythmias. 1 2 3 A CMAP decrease > 40% in the prolonged exercise test is typical. 1 3 Treatment includes acetazolamide and antiarrhythmic agents. 1
